# Enhancing Bottleneck Concept Learning in Image Classification

**DOI:** 10.3390/s25082398

**Published:** 2025-04-10

**Authors:** Xingfu Cheng, Zhaofeng Niu, Zhouqiang Jiang, Liangzhi Li

**Affiliations:** 1 Computer Science Department, Qufu Normal University, Rizhao 276826, China; xingfucheng@qfnu.edu.cn (X.C.); zhaofengniu@qfnu.edu.cn (Z.N.); 2 Osaka University, Osaka 565-0871, Japan; zhouqiang@is.ids.osaka-u.ac.jp

**Keywords:** visual concept, explainable artificial intelligence, image classification

## Abstract

Deep neural networks (DNNs) have demonstrated exceptional performance in image classification. However, their “black-box” nature raises concerns about trust and transparency, particularly in high-stakes fields such as healthcare and autonomous systems. While explainable AI (XAI) methods attempt to address these concerns through feature- or concept-based explanations, existing approaches are often limited by the need for manually defined concepts, overly abstract granularity, or misalignment with human semantics. This paper introduces the Enhanced Bottleneck Concept Learner (E-BotCL), a self-supervised framework that autonomously discovers task-relevant, interpretable semantic concepts via a dual-path contrastive learning strategy and multi-task regularization. By combining contrastive learning to build robust concept prototypes, attention mechanisms for spatial localization, and feature aggregation to activate concepts, E-BotCL enables end-to-end concept learning and classification without requiring human supervision. Experiments conducted on the CUB200 and ImageNet datasets demonstrated that E-BotCL significantly enhanced interpretability while maintaining classification accuracy. Specifically, two interpretability metrics, the Concept Discovery Rate (CDR) and Concept Consistency (CC), improved by 0.6104 and 0.4486, respectively. This work advances the balance between model performance and transparency, offering a scalable solution for interpretable decision-making in complex vision tasks.

## 1. Introduction

Interpreting the behavior of deep neural networks (DNNs) has emerged as a critical challenge in the deployment of these models, particularly in high-stakes domains such as healthcare [1] and autonomous vehicles [2]. Despite their success in achieving state-of-the-art performance, DNNs remain predominantly “black-box” models: their decision-making processes are opaque and difficult to comprehend [3]. This lack of interpretability hinders trust and impedes verification, making it challenging to ensure model reliability in safety-critical applications [4]. In sensor-based systems, such as those employed in autonomous vehicles, medical imaging, and environmental monitoring, the demand for explainable AI is particularly critical. These systems rely extensively on sensor data to make real-time decisions that directly affect human safety. Explainable AI (XAI) [5,6] offers a promising solution by providing transparency through per-pixel relevance information, thereby elucidating the basis for model decisions.

A substantial body of XAI research has concentrated on providing feature-level explanations, particularly at the pixel or patch level for vision-related tasks [7]. These methods assign relevance scores to input features—such as individual pixels in an image—indicating their significance in the model’s decision-making process. Widely recognized approaches, such as saliency maps [8], Grad-CAM [9], and integrated gradients [10], are commonly employed to generate these feature-based explanations. While these methods offer valuable insights, they are often criticized for being challenging to interpret without domain expertise. The relevance information is typically presented at a very granular level, which can be abstract and unintuitive for non-expert users.

To address this gap, recent advancements in explainable AI (XAI) have introduced concept-based methods that aim to represent model behavior using high-level, human-understandable concepts [11]. These approaches seek to align a model’s reasoning with human cognitive processes by linking model outputs to interpretable concepts, such as objects, attributes, or scenes. By focusing on the relationship between these concepts and the model’s decisions, these methods facilitate more transparent explanations that are easier for humans to comprehend [12]. However, most existing concept-based methods rely on the explicit definition of concepts or supervision, which limits their generalizability and scalability. The need for large quantities of labeled data to predefine concepts or reliance on human expertise in defining meaningful concepts presents significant challenges in real-world applications.

We propose a novel concept-based explainability method, the Enhanced Bottleneck Concept Learner (E-BotCL), which leverages self-supervised learning to address the limitations of traditional concept-based methods. E-BotCL represents images by learning the presence or absence of concepts directly from the target task, without depending on manually defined concepts or external supervision. E-BotCL encourages the model to discover task-relevant, human-interpretable semantic concepts. This self-supervised learning framework enhances E-BotCL’s scalability and explainability, making it suitable for a broad range of applications without the need for manual annotation or domain expertise. Through our proposed framework, we aim to make strides toward more interpretable, reliable, and transparent AI models that can be deployed effectively in real-world scenarios, with a focus on enhancing both the model’s accuracy and the explainability of its decisions.

## 2. Related Works

### 2.1. Explainable AI

Explainable artificial intelligence (XAI) seeks to improve the transparency of decision-making processes in machine learning models [5,8,12,13,14,15,16], such as deep neural networks, in response to the growing demand for interpretability and trustworthiness, particularly in high-risk domains. XAI methods are typically divided into two broad categories [17]: post hoc explanations and intrinsic interpretability.

Post hoc explanation methods aim to provide insights into a model’s decision-making after the model has been trained, without modifying the model itself. This category includes techniques such as saliency maps [8], which highlight regions in the input data (e.g., image pixels) that have the greatest impact on the model’s predictions, and feature attribution methods such as LIME [3] and SHAP [18], which approximate decision boundaries by training interpretable surrogate models on localized data regions. Although post hoc explanations are valuable, they are often limited by their lack of semantic clarity and the risk of misinterpretation, as the relevance maps generated may not always align with human-understandable concepts [19].

In contrast, intrinsic interpretability [20] aims to design models that are interpretable by their very structure. These models are typically simpler, and their decision-making processes are more directly comprehensible. Examples of intrinsically interpretable models include decision trees, linear models, and rule-based systems [21]. However, these models often face a trade-off between interpretability and performance, as they may fail to capture the complexity of data as effectively as more sophisticated, opaque models such deep neural networks. Recent research has focused on improving the balance between performance and interpretability, with some efforts dedicated to developing models that retain high accuracy while maintaining transparent internal processes [22,23].

Despite the progress made in XAI, several challenges remain. A key issue is ensuring the trustworthiness of explanations. Explanations must be not only interpretable but also accurate and consistent with the model’s underlying decision-making process. Moreover, there is an ongoing need for improved tools and metrics to evaluate the quality of explanations, as well as to assess the usability, effectiveness, and potential biases in different XAI methods.

### 2.2. Concept-Based Framework for Interpretability

The concept-based framework for interpretability has emerged as a promising approach to enhancing the transparency of deep learning models, particularly by providing human-understandable explanations for complex decision-making processes [12,24]. In contrast to pixel-based or feature-based methods, which explain decisions at a granular level, the concept-based framework aims to offer high-level, semantically meaningful explanations by associating model predictions with a set of interpretable concepts. This approach is inspired by human cognition, where decisions are often based on abstract concepts that are more comprehensible than raw features.

At its core, the concept-based framework operates by defining a set of concepts that capture significant patterns or structures in the input data. These concepts can either be predefined or learned directly from the data. Predefined concepts often stem from domain knowledge, such as medical terminology in healthcare or object categories in image recognition tasks. In contrast, data-driven methods seek to discover these concepts automatically, typically through unsupervised or semi-supervised learning techniques [25,26,27]. Once the concepts are defined, the model’s decision-making process is articulated in terms of the presence or absence of these concepts, providing a more intuitive explanation for human users.

A prominent structure within this framework is the Concept Bottleneck Model (CBM) [24], which directly links model predictions to concept activations. The CBM introduces a bottleneck layer that forces the model to rely on a limited set of concepts to make decisions. As a result, the decision-making process is tightly coupled with the presence or absence of these predefined concepts, offering a transparent mechanism for interpretation. In this structure, the classifier is trained not on raw input data but on the binary activations of concepts, ensuring that the model’s decisions can be traced back to human-understandable features. This concept has been extended and refined in several studies to handle more complex datasets, such as images and text, with notable success.

Despite the advantages of concept-based frameworks in providing semantically rich explanations, designing an appropriate set of concepts remains a critical challenge. Handcrafting concepts through domain expertise, while ensuring interpretability, can be labor-intensive and may not scale well to large, complex tasks. To address this, recent research has focused on automated concept discovery methods [26,27], where concepts are learned directly from data. Techniques such as clustering, factorization, and self-supervised learning have been employed to uncover meaningful concepts that align with human intuition. For example, in image classification, methods such as attention-based mechanisms and unsupervised feature learning have been used to discover high-level object concepts without requiring manual annotations [12]. However, while these automatically discovered concepts can improve scalability and flexibility, they may not always align with human-understandable semantics, potentially leading to interpretations that are less intuitive or harder to verify [28,29].

Moreover, the semantic gap between learned concepts and human understanding remains a persistent challenge. Although concept-based frameworks aim to provide explanations that are easier for humans to interpret, concepts learned through data-driven methods may still lack the richness and clarity of those defined by human experts. To address this, some studies have proposed hybrid approaches that combine predefined and learned concepts, thereby balancing interpretability with model flexibility [12].

### 2.3. Contrastive Learning

Contrastive learning has become a cornerstone technique in self-supervised representation learning, particularly in the field of computer vision. The core idea behind contrastive learning is to learn discriminative features by minimizing the distance between similar samples while maximizing the distance between dissimilar ones. Early approaches to contrastive learning, such as SimCLR [30] and MoCo [31], have significantly advanced the state of the art by utilizing a large number of negative samples or maintaining a memory bank to sustain informative contrast. However, this reliance on numerous negative samples or memory storage introduces substantial computational overhead and complexity, posing challenges for scalability and efficiency, especially in large-scale settings.

To mitigate these drawbacks, SimSiam [32] introduces a more streamlined approach that eliminates the need for negative pairs altogether. Instead of relying on negative samples, SimSiam employs a stop-gradient mechanism to prevent representation collapse during training, while still enabling the model to learn meaningful features. This approach involves two key components: a projector network that transforms the learned representations into a latent space and a predictor network that attempts to predict the representation of one view from another. Notably, SimSiam’s reliance on positive pairs, which are different augmentations of the same image, demonstrates that negative pairs are not a necessary condition for obtaining effective representations. This finding challenges the traditional paradigm in contrastive learning, offering a more computationally efficient framework for self-supervised learning.

SimSiam’s success largely depends on the stop-gradient mechanism, which prevents trivial solutions, such as the collapse of all embeddings into a single point—a common issue in contrastive learning when negative samples are absent. Empirical evaluations on the ImageNet dataset reveal that SimSiam not only achieves competitive performance when compared to methods such as MoCo v2 [30] and BYOL [33] but also requires fewer hyperparameters and less computational resources. As a result, SimSiam presents an attractive alternative for practitioners seeking efficient self-supervised learning methods. Moreover, its simplicity makes it highly scalable and easier to implement than more complex methods that depend on negative sampling or large memory banks.

In the domain of interpretable AI, SimSiam’s ability to learn structured, high-quality representations is particularly promising for concept-based learning frameworks. Concept-based learning seeks to align model representations with human-interpretable concepts, such as object parts, textures, or semantic categories. Unlike traditional supervised approaches, where concepts must be manually defined or pre-annotated, self-supervised contrastive methods such as SimSiam have the potential to autonomously discover meaningful features from the data. This self-discovery of features not only enhances model interpretability but also offers the potential to bridge the gap between high performance and explainability.Furthermore, recent works [34] have underscored the importance of model interpretability in real-world applications, where transparency is critical to ensuring trust and accountability. Thus, SimSiam’s capacity for learning structured representations could provide valuable insights into how deep learning models can achieve both strong performance and greater transparency.

Despite these compelling advantages, there remain open questions and areas for further improvement. For example, although SimSiam’s reliance on positive pairs reduces the need for negative samples, the method still necessitates careful design of augmentation strategies to ensure the diversity of positive pairs. Furthermore, although SimSiam’s simplicity enhances its computational efficiency, its scalability in highly complex tasks beyond image classification, such as object detection and video processing [35,36], remains to be fully evaluated.

## 3. Model

### 3.1. Preliminary

**Figure 1:**
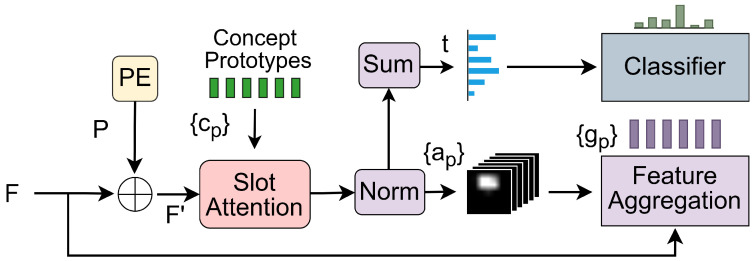
Overview of the concept extractor framework.

Figure 1 provides an overview of the BotCL framework [12] for training concept-based models. The process begins with the input image *x*, from which feature maps *F* are extracted using a backbone convolutional neural network (CNN). These feature maps are subsequently passed to the concept extractor, which performs two critical tasks: it generates the concept bottleneck activation vector *t*, representing the activation probabilities of various visual concepts, and it extracts concept features *G* corresponding to specific regions of interest in the image. The vector *t* is then forwarded to a classifier, which produces the final score *s* for the image classification. Throughout the training, the concept prototypes are constrained using self-supervised and regularization techniques, with both *t* and *G* guiding the learning process.

The concept extractor leverages a slot attention mechanism [37] to identify and extract relevant visual concepts from images. Initially, positional encodings *P* are incorporated into the feature map *F* to preserve spatial information, yielding a modified feature map $F^{\prime }=F+P$. This modified map is then flattened into a 2D tensor of dimensions l×d, where l=hw represents the number of spatial locations, while *d* is the dimensionality of the feature vectors. The slot attention mechanism computes the attention weight $a_{p}$ for each concept *p* across the spatial dimensions, which indicates the spatial distribution of each concept. The features in *F* corresponding to concept *p* are aggregated to form the concept feature $g_{p}$, which is calculated as the attention-weighted average of image features along the spatial dimension.

For classification, a simple fully connected (FC) layer, without any bias terms, is employed. The concept activation vector $t={\left(t_{1},t_{2},\ldots ,t_{k}\right)}^{⊤}$ serves as the input to the classifier, which models the concept bottleneck. Let *M* represent the learnable weight matrix. The predicted class label $\hat{y}$ is computed as

(1) $$\hat{y}=Mt.$$

Here, *M* is the vector corresponding to class *k*, and $M_{kp}$ denotes the *p*th element of this vector. A positive value of $M_{kp}$ suggests that concept *p* frequently co-occurs with class *k* in the dataset, supporting the classification of the image as belonging to class *k*. Conversely, a negative value of $M_{kp}$ implies that concept *p* rarely co-occurs with class *k*, offering less support for the classification.

Given the absence of concept labels, a self-supervised learning approach is employed for concept discovery. To address various target tasks, two distinct loss functions are employed: one for learning visual representations and another for capturing relationships between concepts.

**Reconstruction Loss**: The SENN [25] framework adopts an autoencoder-like structure to learn more accurate representations. This structure assumes that the visual elements in an image are tightly connected to their spatial locations, enabling discrete concepts to reconstruct the original image effectively. A reconstruction loss is designed based on this assumption, where the decoder *D* receives the concept activation *t* and reconstructs the image. The reconstruction loss is formulated as

(2) $$l_{\mathrm{rec}}=\frac{1}{\left|B\right|}\sum_{x\in B}{∥D\left(t\right)-x∥}^{2},$$

where |B| is the mini-batch of images.

**Contrastive Loss**: Since the composition of natural images is inherently arbitrary, the information in the concept activations *t* alone may not suffice for accurate reconstruction. To address this, a contrastive loss function is introduced using image-level labels from the target classification task. Let $\hat{t}=2t-1_{k}$ be a vector of ones. If a pair of concept activations $\left(\hat{t},{\hat{t}}^{\prime }\right)$ corresponds to the same class (i.e., $y=y^{\prime }$, where *y* and $y^{\prime }$ are the labels corresponding to $\hat{t}$ and ${\hat{t}}^{\prime }$, respectively), they are expected to be similar, as the images should share a similar set of concepts. Conversely, if they belong to different classes, the activations should be dissimilar. The contrastive loss is then formulated as

(3) $$l_{\mathrm{ret}}=-\frac{1}{\left|B\right|}\sum \alpha \left(y,y^{\prime }\right)\log J(\hat{t},{\hat{t}}^{\prime },y,y^{\prime }),$$

where α is a weight term that adjusts the contribution of each class to the overall loss, addressing class imbalance. The function *J* is defined as

(4) $$J\left(\hat{t},{\hat{t}}^{\prime },y,y^{\prime }\right)=\left\{\begin{array}{cc} \sigma ({\hat{t}}^{⊤}{\hat{t}}^{\prime }) & \mathrm{for}\;y=y^{\prime }\\ 1-\sigma ({\hat{t}}^{⊤}{\hat{t}}^{\prime }) & \mathrm{otherwise} \end{array}\right.$$

A concept regularizer is introduced to constrain the concept prototypes $\left\{c_{p}\right\}$ and their corresponding features $\left\{g_{p}\right\}$. This regularizer ensures that each concept is stable across images, particularly when $t_{p}$ is close to 1. The consistency loss is defined using cosine similarity as

(5) $$l_{\mathrm{con}}=-\frac{1}{p}\sum_{p}\sum_{g_{p},g_{p}^{\prime }}\left(\mathrm{sim}\left(g_{p},g_{p}^{\prime }\right)\frac{1}{|H_{p}\left|(\right|H_{p}\left|-1\right)}\right),$$

where the second summation iterates over all concept features within the set $H_{p}$, ensuring similarity between features for similar concept activations.

To ensure diversity among concepts, a diversity loss term is introduced. This encourages each concept to correspond to distinct visual elements. The diversity loss is formulated as

(6) $$l_{\mathrm{dis}}=\sum_{p,p^{\prime }}\left(\mathrm{sim}\left(\overset{-}{g_{p}},\overset{-}{g_{p^{\prime }}}\right)\frac{1}{p(p-1)}\right),$$

where the summation is taken over all pairs of concepts, ensuring that different concepts correspond to different visual features.

Finally, a quantization loss is introduced to enforce binarization of the concept activation vector *t*. This loss ensures that the activation values are close to 0 or 1, which is beneficial for interpretability:

(7) $$l_{\mathrm{qua}}=\frac{1}{p|B|}\sum_{x\in B}{∥\mathrm{abs}\left(\hat{t}\right)-1_{p}∥}^{2}$$

where abs(·) represents the element-wise absolute value operation, while ∥·∥ denotes the Euclidean norm.

For the target classification task, the softmax cross-entropy loss, denoted as $l_{\mathrm{cls}}$, is applied. The overall loss function, $L_{base}$, combines the classification loss with various regularization terms:

(8) $$L_{\mathrm{base}}=l_{\mathrm{cls}}+\lambda_{R}l_{R}+\lambda_{\mathrm{con}}l_{\mathrm{con}}+\lambda_{\mathrm{dis}}l_{\mathrm{dis}}+\lambda_{\mathrm{qua}}l_{\mathrm{qua}},$$

where $l_{R}$ is either $l_{\mathrm{rec}}$ or $l_{\mathrm{ret}}$, depending on the target domain, and $\lambda_{\mathrm{qua}}$, $\lambda_{\mathrm{con}}$, $\lambda_{\mathrm{dis}}$, and $\lambda_{R}$ are the regularization coefficients that balance the contributions of each term.

### 3.2. E-BotCL

**Figure 2:**
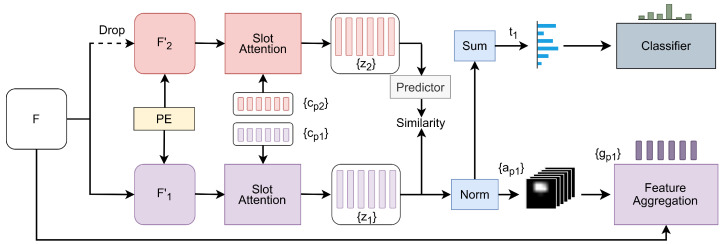
Overview of the contrastive concept extractor framework.

E-BotCL is an enhanced iteration of the original BotCL framework [12], designed to improve concept discovery and classification robustness by integrating a dual-path contrastive learning strategy, inspired by SimSiam [32]. Given a dataset $S=\{\left(x_{i},y_{i}\right)|i=1,2,\ldots ,N\}$, where $x_{i}$ represents an image and $y_{i}$ is the target class label associated with $x_{i}$ from the set Ω. Figure 2 details the architecture of the Contrastive Concept Extractor, where PE denotes position embedding.

Given an input image *x*, the backbone convolutional neural network *B* extracts a feature map $F=B\left(x\right)\in R^{d\times h\times w}$. This feature map *F* is then passed through the Contrastive Concept Extractor $e_{C}$, where *C* is a matrix whose *p*th column vector $c_{p}$ represents a learnable concept prototype. The Contrastive Concept Extractor produces a concept bottleneck activation $t_{p}\in {\left[0,1\right]}^{l}$, indicating the presence of each concept, as well as concept features $G\in R^{d\times p}$ corresponding to the regions where each concept is present. The concept activation $t_{1}$ is subsequently used as input to the classifier to compute the classification score $s\in {\left[0,1\right]}^{\left|\Omega \right|}$.

### 3.3. Contrastive Concept Extractor

The feature map *F* is first processed through a 1×1 convolutional layer to project it into a latent space, followed by batch normalization and ReLU activation. This operation yields the base feature representation:

(9) $$F_{i}=\mathrm{ReLU}\left(\mathrm{Norm}\left({\mathrm{Conv}}_{1\times 1}\left(F\right)\right)\right).$$

Inspired by Siamese networks, the model employs two augmented views ($F_{1}$, $F_{2}$) of $F_{i}$ for self-supervised contrastive learning: Branch 1 retains the original features $F_{1}$. Branch 2 applies stochastic dropout (simsiam_drop) to $F_{2}$ as a form of feature augmentation.

Both feature representations are then combined with position embeddings and reshaped into sequential features:

(10) $$F_{i}^{\prime }=\mathrm{Reshape}\left(F_{i}+P\right),\;i\in \left\{1,2\right\}.$$

The slot attention mechanism [37,38] is employed to compute the spatial attention of concept *p* between $c_{pi}$ and $F_{i}^{\prime }$. Let $Q\left(c_{pi}\right)\in R^{d}$ and $K\left(F_{i}^{\prime }\right)\in R^{d\times l}$ represent the nonlinear transformations of $c_{pi}$ and $F_{i}^{\prime }$, respectively. These transformations are implemented using multilayer perceptrons (MLPs) composed of three fully connected (FC) layers with ReLU activation between them. The attention $a_{p1}\in {\left[0,1\right]}^{l}$ is computed using a regularization function φ as follows:

(11) $$a_{p1}=\varphi \left(Q{\left(c_{p1}\right)}^{⊤}K\left(F_{1}^{\prime }\right)\right).$$

This attention mechanism identifies the spatial location of concept *p* in the image. If concept *p* is absent, the corresponding entries of $a_{p1}$ remain close to zero. To quantify the presence of each concept, we compute the concept activation score $t_{p}$ by aggregating the spatial dimension of $a_{p1}$ as $t_{p}=\tanh\left(\sum_{n}a_{p1_{n}}\right)$, where $a_{p1_{n}}$ is the *n*th element of $a_{p1}$.

### 3.4. Slot-Based Feature Aggregation

During training, we aggregate the features in *F* corresponding to concept *p* into the concept feature $g_{p1}$, as follows:

(12) $$g_{p1}=Fa_{p1},$$

which provides the weighted average of the image features in the spatial dimension, with attention applied.

### 3.5. E-BotCL Loss

The following pseudocode outlines the process for calculating the contrastive learning loss in a PyTorch-like framework (Algorithm 1).

The slot-updated prototypes $z_{1}$ and $z_{2}$ are passed through a prediction network *h*, which projects them into a shared representation space. To align the cross-branch representations, a negative cosine similarity loss function is employed. This is expressed as

(13) $$L_{\mathrm{cont}}=1-\frac{1}{2}\left[D\left(p_{1},z_{2}\right)+D\left(p_{2},z_{1}\right)\right],$$

where D(p,z) represents the negative cosine similarity measure, $p_{1}=h\left(z_{1}\right)$ and $p_{2}=h\left(z_{2}\right)$ are the predictions for $z_{1}$ and $z_{2}$ obtained from the prediction network *h*, and $z_{1}$ and $z_{2}$ are the slot-updated prototypes. The operation *D* computes the cosine similarity between the predictions and the stop-gradient versions of the prototypes (i.e., $z_{1}$ and $z_{2}$ are detached during the loss calculation). The loss function aims to maximize the similarity between the projected representations of $z_{1}$ and $z_{2}$ across different branches by minimizing the negative cosine similarity.

The overall loss is defined as the sum of the contrastive loss and the base loss:

(14) $$L=L_{\mathrm{cont}}+L_{\mathrm{base}}.$$

**Algorithm 1:** Contrastive Loss Pseudocode, PyTorch-like
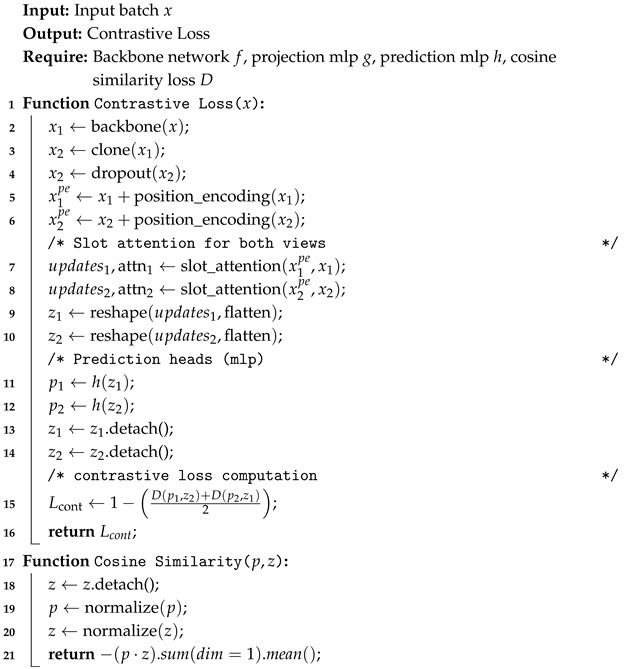


## 4. Results

### 4.1. Experimental Settings

We evaluated E-BotCL on the CUB200 [39] and ImageNet [40] datasets. For both CUB200 (using the same data split as in [24]) and ImageNet, we employed a pre-trained ResNet-18 [41] as the backbone, reducing the channel size from 512 to 128 using a 1 × 1 convolutional layer. We selected p=20 as the number of concepts for both datasets. The images were resized to 256×256 and then cropped to 224×224. During training, random horizontal flipping was applied as the sole data augmentation technique. The weights for each loss term were set to the default values: $\lambda_{qua}=0.1$, $\lambda_{con}=0.01$, $\lambda_{dis}=0.05$, and $\lambda_{R}=0.1$. The learning rate was set to 0.0001, the number of epochs was set to 60, and the batch size was set to 128.

### 4.2. Interpretability

**Figure 3:**
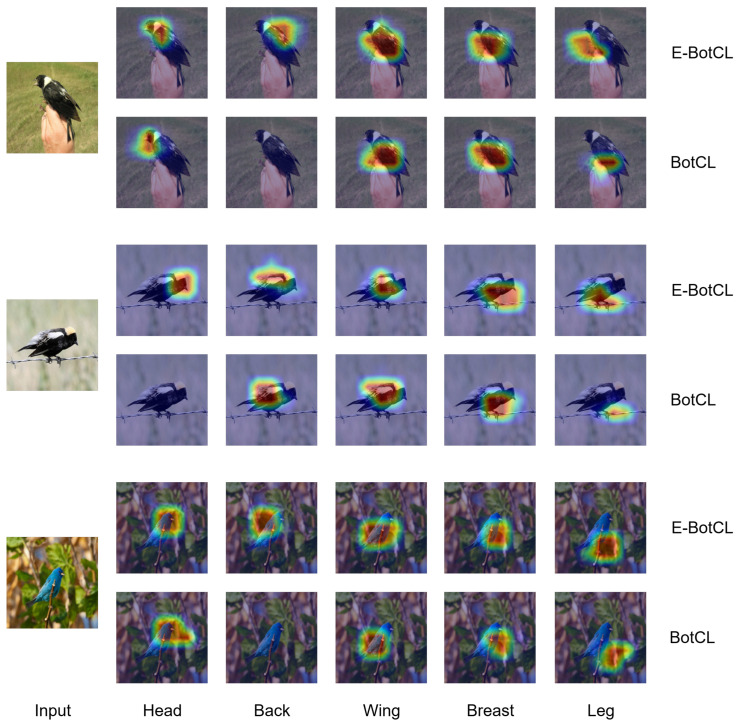
Visualization comparison of five key body parts between BotCL and E-BotCL on the same input image.

Figure 3 visually contrasts the top five salient concepts identified by the E-BotCL and BotCL models in bird image recognition through heatmap analysis. This comparison highlights the differences between the two models in terms of interpretability and conceptual accuracy. From the perspective of concept identification, E-BotCL demonstrates a qualitative improvement over BotCL. Specifically, E-BotCL achieves finer granularity in locating key features within the image. For instance, in the recognition of a bird’s head and back, the heatmap produced by E-BotCL shows highly focused activation areas, distinctly separating these two features. In contrast, BotCL often exhibits numerous regions with no concept activation. Moreover, BotCL’s concept activation map sometimes confuses the leg region with the background or abdomen, whereas E-BotCL correctly identifies the legs as distinct concepts, forming well-defined attention regions. This ability to capture spatially separated yet semantically related concepts significantly enhances the interpretability of the model’s decision-making process.

**Figure 4:**
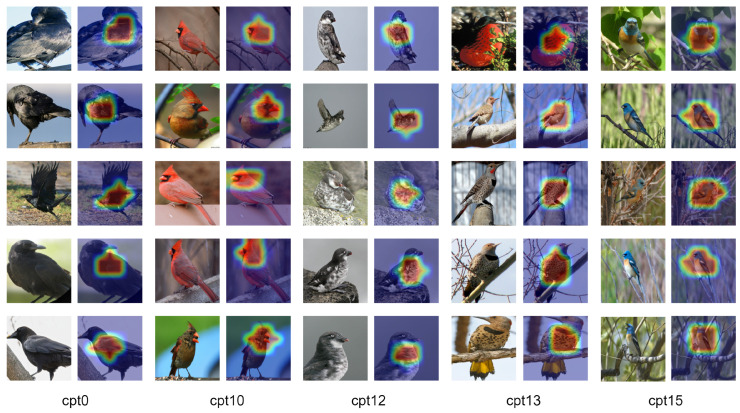
Examples of activated concepts learned from the CUB200 dataset.

In addition, Figure 4 shows examples of concept activations learned by E-BotCL on the CUB200 dataset, further illustrating the model’s capacity for fine-grained interpretability. E-BotCL not only distinguishes between various body parts of a bird as independent concepts (e.g., cpt0 representing the bird’s wings and cpt10 representing the bird’s head) but also identifies more subtle and intricate patterns present on the bird’s body. For example, the concepts activated for the bird’s spots (cpt12 and cpt13) and the stripes on its wings (cpt15) are clearly visible in the figure, reflecting the model’s ability to recognize and isolate fine-grained features that are crucial for concept explanation. These results emphasize the enhanced granularity and flexibility of the E-BotCL framework in learning both the broader structural components and the finer texture-based details, which contributes significantly to improving the model’s ability to explain concepts.

By effectively capturing both high-level body parts and low-level texture patterns, E-BotCL not only enhances the transparency of the decision-making process but also strengthens the model’s ability to provide clear and interpretable explanations for the concepts it identifies.

**Figure 5:**
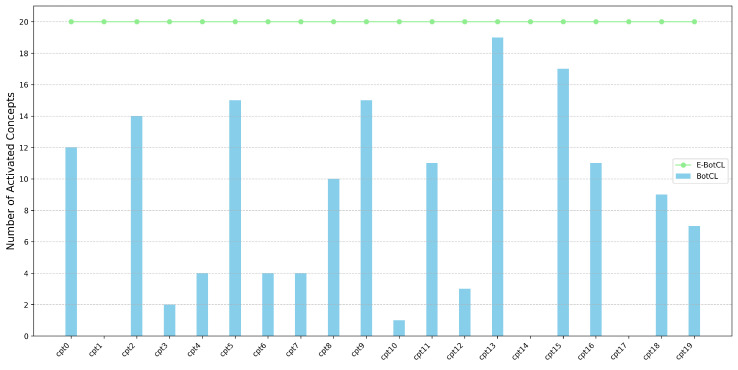
Concept activation status within each concept.

We set the number of concepts to 20 and selected the top 20 activated samples for each concept to analyze the activation patterns. The experimental results clearly demonstrate that all concepts in the E-BotCL method exhibited activation, as illustrated in Figure 5. This indicates a more consistent and robust activation across concepts than in the BotCL method, which showed notable shortcomings. Specifically, BotCL failed to activate samples for concepts cpt1, cpt14, and cpt17. Moreover, the activation distribution for the remaining concepts in BotCL was sparse, with concept cpt3 having only two activated samples; concepts cpt4, cpt6, cpt7, and cpt10 having four each; and concept cpt12 having three activated samples. This overall scarcity of activated samples suggests that the BotCL method struggles to generate meaningful and well-represented concepts, resulting in a less effective concept activation process.

**Figure 6:**
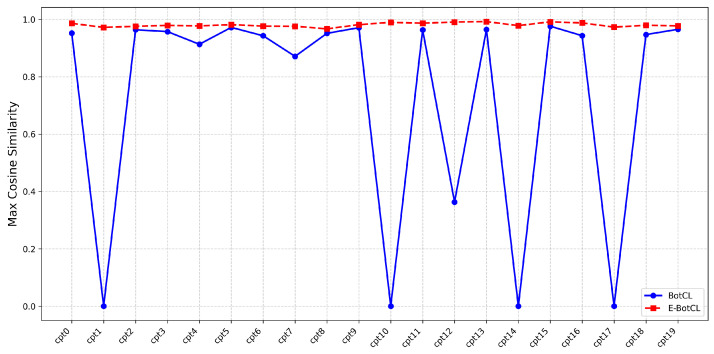
Maximum cosine similarity within each concept category.

To further assess the concept explanation performance, we quantitatively compared the internal similarity within each concept for both E-BotCL and BotCL. Higher internal similarity indicates better alignment and coherence within the concept. As shown in Figure 6, the internal similarity for the concepts generated by E-BotCL was consistently superior to that of BotCL, signifying that E-BotCL produces more coherent and tightly defined concepts. This higher similarity is indicative of the method’s ability to capture more accurate and consistent concept representations, which is essential for interpretability in model decision-making.

Additionally, we evaluated the degree of independence between concepts, where a lower independence value indicates a higher degree of overlap and interaction between concepts—often a desirable characteristic in complex models that aim to reflect real-world semantic relationships. The Distinctiveness Average Similarity (DAS) for E-BotCL was 0.592, outperforming the 0.578 achieved by BotCL. This observed difference can be attributed to the fact that BotCL generates certain meaningless concepts that do not exhibit strong activation patterns or meaningful relationships with other concepts, leading to lower internal similarity and higher independence. In contrast, E-BotCL produces concepts that, while distinct, demonstrate a degree of overlap in their activations. This overlap suggests that the concepts in E-BotCL are more semantically coherent and interrelated, which ultimately leads to the observed higher overall similarity.

### 4.3. Classification Performance

We conducted a comprehensive comparison of the performance of E-BotCL with BotCL, k-means clustering, Principal Component Analysis (PCA) (re-implemented from [12,16]), and other leading concept-based models. The results are summarized in Table 1. Notably, E-BotCL outperforms all baseline methods, achieving the highest accuracy on both the CUB200 and ImageNet datasets. This reinforces our hypothesis that contrastive self-supervision plays a pivotal role in facilitating effective concept discovery, providing both interpretability and robustness to the learned representations.

**Figure 7:**
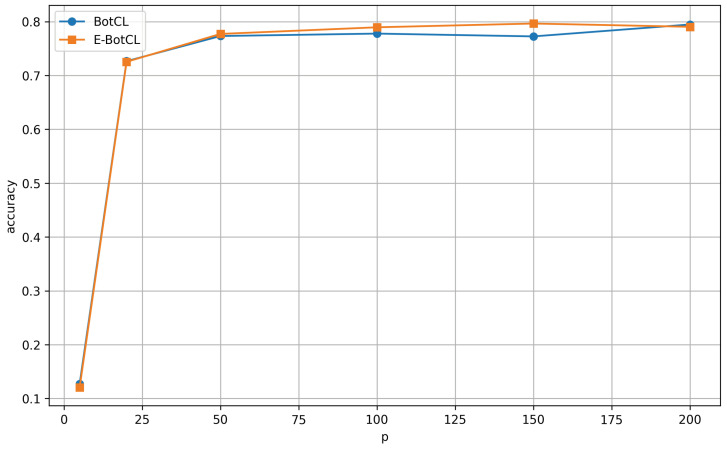
Impact of the number of concepts (*p*) on BotCL and E-BotCL classification accuracy.

To further understand the relationship between the number of concepts and classification accuracy, we explored this dynamic for both E-BotCL and BotCL on the CUB200 dataset. As depicted in Figure 7, E-BotCL maintains strong performance when the number of concepts is between 20 and 200. This range demonstrates that the method is capable of adapting well to datasets of varying sizes, consistently delivering competitive accuracy even for smaller to medium-sized concept sets. In particular, E-BotCL excels in situations where the number of concepts is neither too small nor too large, offering a balance that ensures high-quality concept learning.

On the other hand, when the number of concepts is either below 20 or above 200, BotCL emerges as the superior model. These observations suggest that, while E-BotCL is robust within an optimal concept range, BotCL may be more effective in scenarios that involve either a very small or a very large number of concepts. This indicates that the effectiveness of concept-based learning approaches is significantly influenced by the scale and distribution of concepts, with E-BotCL showing particular promise for moderate ranges. These results collectively confirm that the dual-path contrastive learning strategy employed by E-BotCL contributes significantly to both concept discovery and classification performance.

### 4.4. User Study

The user study aims to evaluate the performance of E-BotCL in human interpretability using real-world datasets. Participants were tasked with observing test images annotated with concept attention maps and selecting the phrase from a predefined vocabulary that most accurately describes the concept (i.e., the attended region). If no consistent visual element could be identified, participants were allowed to choose “none”. For each concept in the CUB200 dataset, 20 participants were recruited for evaluation. For both E-BotCL and BotCL, we selected 200 concept attention maps for each method to be used in the user evaluation.

Table 2 compares the performance of two methods, BotCL and E-BotCL, across three key metrics [12]: **Concept Discovery Rate (CDR)**, **Concept Consistency (CC)**, and **Mutual Information between Concepts (MIC)**. These metrics were selected to provide a comprehensive assessment of the methods’ effectiveness in concept learning and interpretability, particularly in terms of their ability to discover and express concepts.

CDR measures the proportion of participants who successfully identify and generalize visual elements as valid concepts. A higher CDR indicates that participants are better at recognizing consistent and representative visual features from the data, thereby forming clearer concepts. E-BotCL performs exceptionally well in terms of CDR, suggesting that the method is highly consistent in the concept discovery process, with all participants successfully identifying and generalizing the concept. In contrast, BotCL shows significant variability in its CDR, indicating that BotCL has unstable performance in concept discovery and struggles to provide consistent visual feature guidance for all participants.

CC quantifies the degree of agreement between participants in their expressions of the same concept, reflecting the method’s effectiveness in guiding participants toward a consistent understanding of the concept. A high CC value suggests that different participants use similar language and terminology to describe the same concept, indicating that the concept is both clear and stable. The experimental results reveal that E-BotCL achieves a CC mean of 0.6952 with a standard deviation of 0.1396, demonstrating its ability to effectively guide participants toward a highly consistent conceptual understanding, with good stability across different participants. In contrast, BotCL’s CC is 0.2466 with a standard deviation of 0.3361, showing considerable fluctuation and highlighting its limitations in ensuring concept consistency, with substantial variation in participants’ understanding.

MIC reflects the similarity of response distributions between different concepts, with lower values indicating greater differentiation between concepts and avoidance of overlap. For an effective concept learning method, the MIC should be as low as possible to ensure that each concept remains sufficiently distinct. E-BotCL excels in MIC, indicating that it effectively minimizes redundancy between concepts, preventing excessive overlap. In contrast, BotCL’s MIC suggests some degree of overlap and information redundancy between concepts, leading to poorer differentiation.

Overall, E-BotCL outperforms BotCL on all three metrics, providing further evidence of its superiority in enhancing the quality of concept discovery and learning. Specifically, in the context of interpretability and model transparency, E-BotCL better supports model explainability, ensuring that the learned concepts not only exhibit high consistency in expression but also offer clearer and more distinguishable representations.

### 4.5. Ablation Study

The ablation study presented in Table 3 examines the impact of different components of the E-BotCL framework on the CUB200 dataset. Specifically, we evaluate the inclusion of Concept Learning (CL) and Multi-Task Loss (MTL) alongside the baseline BotCL approach in terms of accuracy, model complexity (number of parameters), training time, and GPU memory consumption.

From the results, we observe that the baseline BotCL model achieves an accuracy of 0.7733 with a parameter count of 14.37 M. Introducing the CL component improves accuracy to 0.7758 but comes with an increase in model complexity (15.61 M parameters) and a rise in training time from 65 to 83 min. Similarly, adding the MTL component to BotCL results in an accuracy of 0.7765 while further increasing the parameter count to 16.10 M and requiring 96 min for training. The full E-BotCL model, which incorporates both CL and MTL, achieves the highest accuracy (0.7772). However, this comes at the cost of additional computational demands, with a parameter count of 17.34 M, a training time of 107 min, and increased GPU memory consumption of 9.6 GB.

These results indicate that both CL and MTL contribute to performance improvements, albeit at the expense of higher computational costs. The incremental accuracy gains suggest that the inclusion of these components enhances the model’s interpretability and robustness without significantly compromising efficiency. Therefore, the full E-BotCL framework represents a balanced trade-off between accuracy and computational resources, making it a viable approach for interpretable image classification tasks.

## 5. Conclusions

This study addresses the critical challenge of balancing model performance and interpretability in deep learning by introducing the Enhanced Bottleneck Concept Learner (E-BotCL). By integrating self-supervised contrastive learning, attention mechanisms, and multi-task regularization, E-BotCL autonomously discovers human-interpretable semantic concepts, eliminating the need for manual annotations or predefined concept sets. The dual-path contrastive framework, inspired by SimSiam, facilitates robust concept prototype learning, while the slot-based attention mechanism and feature aggregation strategies ensure precise spatial localization and semantic alignment of the discovered concepts.

Experimental results on the CUB200 and ImageNet datasets demonstrate the superiority of E-BotCL over existing concept-based models, achieving state-of-the-art classification accuracy rates of 72.6% and 77.0%, respectively, while maintaining high interpretability. Notably, E-BotCL excels in concept consistency and distinctiveness, as evidenced by quantitative metrics (e.g., higher intra-concept similarity) and qualitative visualizations (e.g., accurate localization of bird body parts and patterns). These findings underscore the framework’s ability to bridge the semantic gap between low-level features and high-level, human-understandable concepts.

This work significantly advances the practical application of explainable AI in domains that necessitate transparent decision-making, including healthcare, autonomous systems, and sensor-driven technologies. By enhancing the interpretability of deep learning models, particularly within sensor-based applications, our approach contributes to the development of more reliable and trustworthy sensor systems. For instance, in the context of autonomous vehicles, the ability to explain how sensor data (from cameras, LiDAR, and radar) informs decision-making can substantially improve both safety and user trust. Similarly, in medical sensor technologies, offering interpretable AI-driven insights into diagnostic sensor data can empower clinicians to make more informed and accurate decisions. Future research could focus on extending E-BotCL to multimodal tasks, refining concept diversity through adversarial training, or incorporating domain-specific constraints to enhance performance in specialized applications.

## Figures and Tables

**Table 1:** Performance Comparison of Classification Accuracy. The best concept-based method is highlighted in bold. For ImageNet, the top 200 classes were used.

	CUB200	ImageNet
k-means [16]	0.063	0.427
PCA [16]	0.044	0.139
SENN [25]	0.642	0.673
ProtoPNet [42]	0.725	0.752
BotCL	0.725	0.768
E-BotCL	**0.726**	**0.770**

**Table 2:** Results of user study.

	CDR (↑)		CC (↑)		MIC (↓)	
	**Mean**	**Std**	**Mean**	**Std**	**Mean**	**Std**
BotCL	0.3896	0.4527	0.2466	0.3361	0.2489	0.0787
E-BotCL	1.0000	0.0000	0.6952	0.1396	0.1706	0.0512

**Table 3:** Ablation study of E-BotCL components on CUB200 dataset.

BotCL	CL	MTL	Acc	#Params	Training Time	GPU Memory
✓			0.7733	14.37 M	65 min	6.8 GB
✓	✓		0.7758	15.61 M	83 min	7.1 GB
✓		✓	0.7765	16.10 M	96 min	9.3 GB
✓	✓	✓	0.7772	17.34 M	107 min	9.6 GB

## Data Availability

All datasets used in this article are publicly accessible.
